# P-523. Zelicapavir (EDP-938) Antiviral Treatment is Associated with Shortened Duration of RSV Symptoms in a Randomized, Double-Blind, Placebo-Controlled, Clinical Trial in Children 28 Days to 36 Months of Age

**DOI:** 10.1093/ofid/ofaf695.738

**Published:** 2026-01-11

**Authors:** Christopher E Harris, John DeVincenzo, Stephen Huang, Shijie Chen, Taylor Ngo, Alaa Ahmad, Scott T Rottinghaus

**Affiliations:** Enanta Pharmaceuticals, Watertown, MA; Enanta Pharmaceuticals, Watertown, MA; Enanta Pharmaceuticals, Inc., Watertown, Massachusetts; Enanta Pharmaceuticals, Watertown, MA; Enanta Pharmaceuticals, Watertown, MA; Enanta Pharmaceuticals, Watertown, MA; Enanta Pharmaceuticals, Watertown, MA

## Abstract

**Background:**

RSV causes significant morbidity in young children and high risk adults. Zelicapavir (EDP-938) is an oral, non-nucleoside, small molecule N-protein inhibitor in development to treat RSV. Here we present further analyses of clinical RSV symptoms from the first pediatric trial.

**Figure 1.**
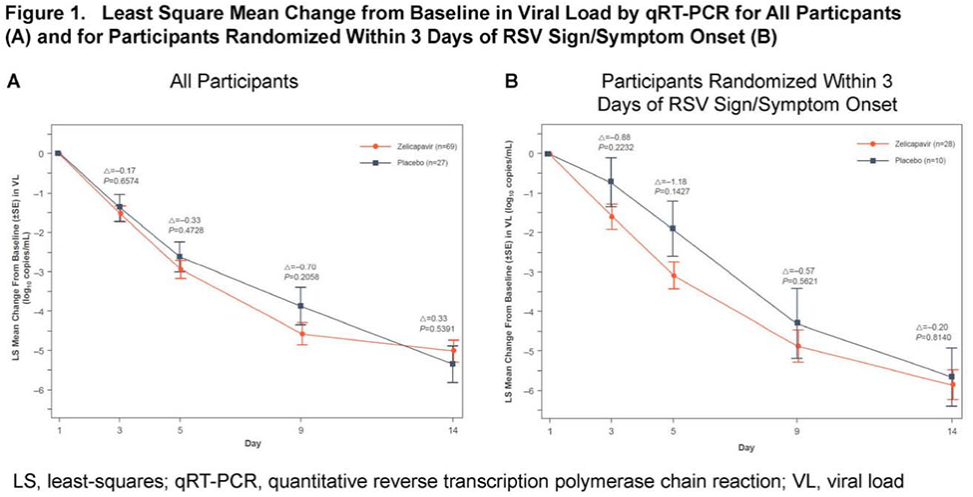
Least Square Mean Change from Baseline in Viral Load by qRT-PCR for All Particpants (A) and for Participants Randomized Within 3 Days of RSV Sign/Symptom Onset (B)

**Figure 2.**
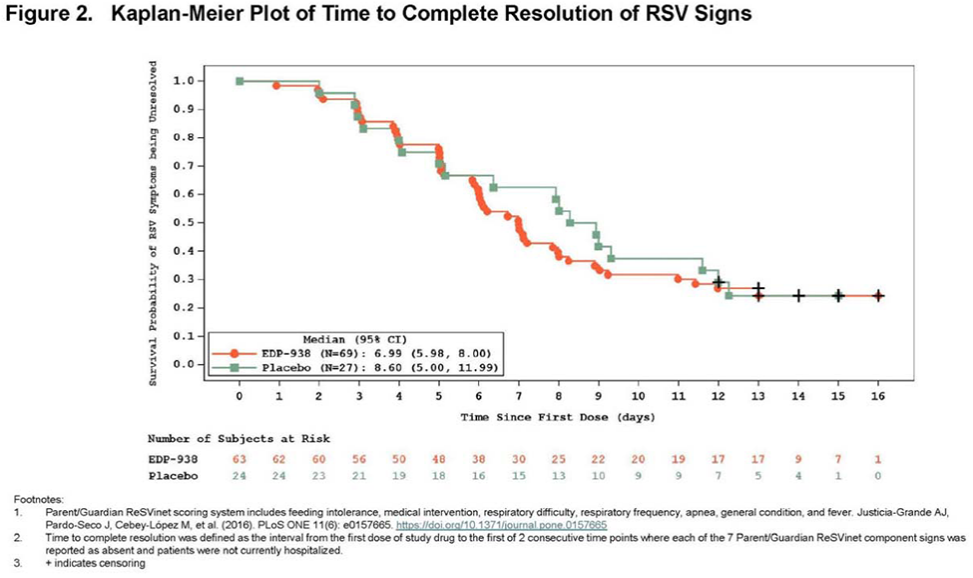
Kaplan-Meier Plot of Time to Complete Resolution of RSV Signs

**Methods:**

A randomized, double-blind, placebo (pbo)-controlled international trial was conducted in children 28 days to 36 months of age evaluating the safety, PK, and antiviral activity of zelicapavir given once daily for 5 days (NCT04816721). Caregivers reported the severity of RSV-related symptoms daily from baseline through Day 14 using the Parent/Guardian ReSVinet clinical scoring system^1^ as an exploratory assessment.

**Figure 3.**
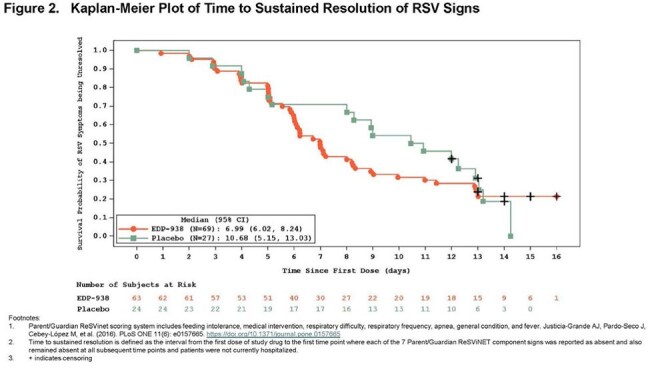
Kaplan-Meier Plot of Time to Sustained Resolution of RSV Signs

**Results:**

96 patients were randomized and dosed (69 zelicapavir; 27 pbo). Greater viral load reduction was observed in zelicapavir recipients, with a decline of 1.18 log_10_ at Day 5 vs. pbo in a prespecified analysis of children randomized within 3 days of RSV symptom onset (Fig 1).

Prespecified ReSVinet analysis showed no difference between treatment and placebo in time to resolution of symptoms (defined as mild or absent). An ad hoc analysis of time to complete resolution of symptoms (defined as absent and discharged from hospital), showed an estimated Kaplan-Meier median (95% confidence interval) time of 6.99 days (5.98-8.00) for zelicapavir vs. 8.60 days (5.00-11.99) for pbo (Fig 2). Similarly, an analysis of sustained resolution (defined as absent and remaining absent at all subsequent time points and discharged from hospital) with times of 6.99 days (6.02-8.24) for zelicapavir vs. 10.68 days (5.15-13.03) for pbo (Fig 3).

All zelicapavir recipients achieved model-predicted target drug exposures. Adverse events were similar between treatment groups, with none leading to treatment discontinuation or study withdrawal.

**Conclusion:**

Zelicapavir treatment was associated with a shorter time to complete resolution of RSV-related symptoms assessed by Parent/Guardian ReSVinet in an ad hoc analysis of a randomized trial for children 28 days to 36 months of age, supporting further trials in pediatric RSV infection.

**Disclosures:**

Christopher E. Harris, MD, Enanta Pharmaceuticals: Employee|Enanta Pharmaceuticals: Stocks/Bonds (Private Company) John DeVincenzo, MD, Enanta Pharmaceuticals: Employee of Enanta Pharmaceuticals|Enanta Pharmaceuticals: Stocks/Bonds (Public Company) Stephen Huang, M.D., Enanta Pharmaceuticals, Inc.: Employee|Enanta Pharmaceuticals, Inc.: Stocks/Bonds (Public Company) Shijie Chen, PhD, Enanta Pharmaceuticals: Employee of Enanta Pharmaceuticals|Enanta Pharmaceuticals: Stocks/Bonds (Public Company) Taylor Ngo, MPH, Enanta Pharmaceuticals: Employee|Enanta Pharmaceuticals: Stocks/Bonds (Private Company) Alaa Ahmad, PhD, Enanta Pharmaceuticals: Employee of Enanta Pharmaceuticals|Enanta Pharmaceuticals: Stocks/Bonds (Public Company) Scott T Rottinghaus, MD, Enanta Pharmaceuticals: Employee of Enanta Pharmaceuticals|Enanta Pharmaceuticals: Stocks/Bonds (Public Company)

